# Peer Review of “Willingness to Pay for the COVID-19 Vaccine and Its Correlates in Bangladesh: Cross-Sectional Study”

**DOI:** 10.2196/79354

**Published:** 2025-08-15

**Authors:** Enamul Hoque

**Affiliations:** 1 The University of Western Australia, 35 Stirling Highway, Perth, Australia, 61 86488 60

**Keywords:** Bangladesh, willingness to pay, vaccines, COVID-19, infectious diseases, infection control, public health, public safety, cross-sectional study, financial

*This is a peer-review report for “Willingness to Pay for the COVID-19 Vaccine and Its Correlates in Bangladesh: Cross-Sectional Study.”*

## Round 1 Review

### Minor Comments

1. In lines 79 and 80 of the manuscript [1], it is confusing why this wouldn’t be considered nationally representative if the data collection was conducted online.
2. As around 50% of the people are not interested in paying for the vaccine, this result should be considered with caution.
